# P25 A quality improvement initiative to improve IV gentamicin management and promote a multidisciplinary approach to patient-centred care in the acute setting

**DOI:** 10.1093/jacamr/dlaf118.032

**Published:** 2025-07-14

**Authors:** H Qadri, R Rodger, S Thompson, A Gallagher, E Simpson, F Di Bonar, M Joyce

**Affiliations:** Royal Alexandra Hospital, Paisley, UK; Royal Alexandra Hospital, Paisley, UK; Royal Alexandra Hospital, Paisley, UK; Royal Alexandra Hospital, Paisley, UK; Royal Alexandra Hospital, Paisley, UK; Royal Alexandra Hospital, Paisley, UK; Royal Alexandra Hospital, Paisley, UK

## Abstract

**Background:**

Gentamicin is an aminoglycoside antibiotic with a narrow therapeutic index requiring therapeutic drug monitoring (TDM) to optimize dosing and minimize patient risk of renal and ototoxicity.^1^ In NHS Greater Glasgow and Clyde (GGC), to improve safety and patient-centred care IV gentamicin is prescribed using specific prescribing, administering and monitoring (PAM) charts, restricted to 4 days treatment and patient information leaflets (PILs) provided.^2,^ Keeping patients informed and involved^3^ when prescribing aminoglycosides supports early detection and management of adverse effects such as ototoxicity that can otherwise be more difficult to recognize. A recent gentamicin quality improvement (QI) baseline evaluation at the Royal Alexandra Hospital (RAH)^4^ identified targeted areas for improvement including provision of counselling and PILs to patients, administration documentation on the electronic prescribing system (HEPMA), dose delays, TDM sampling, and documentation of 48 hourly prescribing. Unclear responsibility, outdated PAM charts without PILs, and lack of time and training, were identified as barriers to improving IV gentamicin multidisciplinary management at RAH.^4^

**Objectives:**

To apply QI methodology to improve the multidisciplinary management of IV gentamicin at the RAH in line with QI targets identified from baseline evaluation.

**Methods:**

Standardized Microsoft Forms^4^ and HEPMA reports were used to collect repeat baseline (2023) and post-change (Jan-Feb and Oct-Nov 2024) data in RAH adult medical, surgical and elderly care inpatients prescribed IV gentamicin, prophylactic gentamicin was excluded. HEPMA prescriptions and PAM charts were prospectively reviewed and quantitative data collated (Excel) for comparison with QI targets. QI ‘tests of change’ included: ‘bite-size’ multidisciplinary ward teaching, introduction of a pharmacy technician gentamicin counselling service,^5^ collation of qualitative patient feedback, ‘Gold Star’ awards, multidisciplinary staff champions, ‘Top Tips’ IV gentamicin poster development and prospective audit/feedback.

**Results:**

Repeat baseline data (*n*=97, 46% male, mean age 66 years) aligned with previously met targets (Figure 1) and areas requiring improvement (Figure 2). In total 129 patients (41% male, mean age 63 years) were reviewed post-change. In both groups the most common indications for IV gentamicin were intra-abdominal sepsis and urinary sepsis. Post-change although the only QI target met was for removal of outdated PAM charts, a number of improvements were achieved including: increased delivery of counselling and PILs from 4% to 21%, appropriate 48 hourly prescribing documentation increased from 58% to 85%, and IV gentamicin doses given on time increased from 75% to 88%. Administration documentation on HEPMA (41%), and initial gentamicin blood samples taken at the appropriate time (55%), improved marginally from baselines of 38% and 51%, respectively (Figure 2). Qualitative patient feedback highlighted the importance of providing a patient-centred care approach to IV gentamicin management in hospital.

**Conclusions:**

Using QI methodology to promote a multidisciplinary approach to IV gentamicin management resulted in improvements in delivery of patient counselling, delayed doses, TDM and prescribing and administration documentation. This is important in terms of patient safety, antimicrobial dose optimization and stewardship and embedding a patient-centred care approach to IV gentamicin management in the acute setting. Further QI work is necessary to maintain and optimize improvements to meet QI targets.

**Figure 1.**
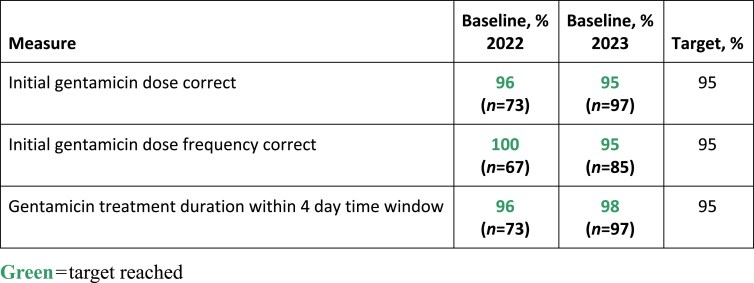
Quantitative data measures at baseline.

**Figure 2.**
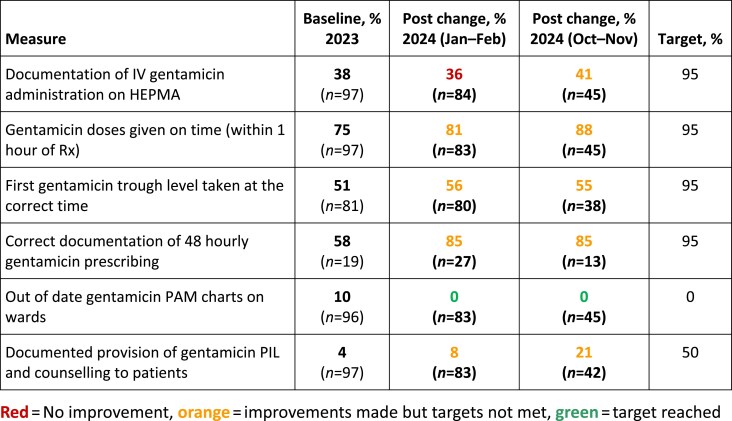
Quantitative data measures at baseline and post ‘tests of change’ and targets.
